# Application of Solid-State NMR to Reveal Structural Differences in Cefazolin Sodium Pentahydrate From Different Manufacturing Processes

**DOI:** 10.3389/fchem.2018.00113

**Published:** 2018-04-10

**Authors:** Ye Tian, Wei D. Wang, Wen-Bo Zou, Jian-Qin Qian, Chang-Qin Hu

**Affiliations:** ^1^National Institutes for Food and Drug Control, Beijing, China; ^2^State Key Laboratory of Applied Organic Chemistry, College of Chemistry and Chemical Engineering, Lanzhou University, Lanzhou, China; ^3^Zhejiang Institute for Food and Drug Control, Hangzhou, China

**Keywords:** cefazolin sodium, conformation, polymorphism, solid-state NMR, solvate

## Abstract

Solid-state Nuclear magnetic resonance, thermogravimetric analysis, X-ray diffraction, and Fourier-transform infrared spectroscopy were combined with theoretical calculation to investigate different crystal packings of α-cefazolin sodium obtained from three different vendors and conformational polymorphism was identified to exist in α-cefazolin sodium. Marginal differences observed among cefazolin sodium pentahydrate 1, 2, and 3 were speculated as being caused by the proportion of conformation **2**.

## Introduction

Understanding the solid form of an active pharmaceutical ingredient (API) is a key part of its development. The differential and specific arrangements of molecules, such as co-crystals, solvates, polymorphs, and hydrates can determine a new chemical entity's crystal form. The mechanical and physical characteristics of pharmaceutical powders and the manufacturing process depend on their crystal morphology and structure, which are basically determined by the directionality and strength of interactions among molecules within the crystal lattice (Hancock et al., 2000). For organic crystals, hydrogen-bonding frameworks are often formed among molecules. Hydrogen bonding has received special focus because its directional nature causes anisotropy in molecular crystals (Payne et al., 1996). Therefore, the types and numbers of donors and acceptors of hydrogen bonds and the directionality of molecular interactions should have a large effect on crystal structures.

**Figure 1:**
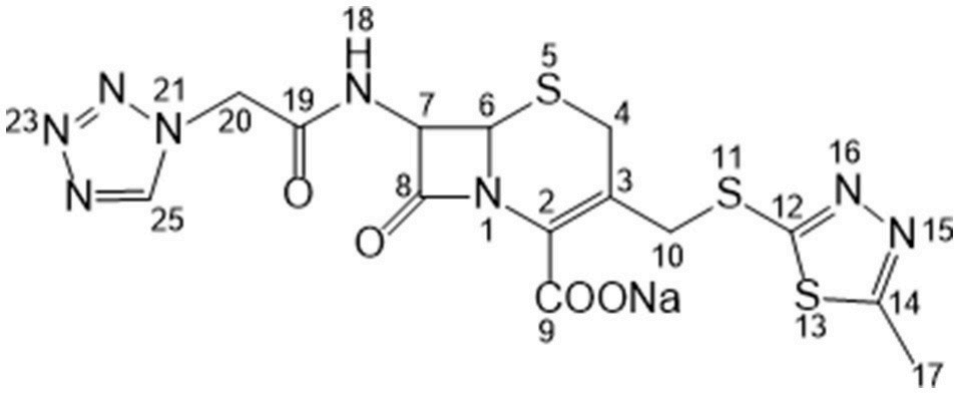
Structure of cefazolin sodium showing the numbering of the carbons.

Cefazolin sodium (hereafter referred to as CEZ-Na) is a first-generation cephalosporin antibiotic (Figure 1). It has been widely used for treating various bacterial infections of the respiratory tract, skin, skin structure, bone and joints, and genital, urinary, and biliary tracts since the early 1970s (Nishida et al., 1969; Kariyone et al., 1970; Kusaba, 2009). It exists in four crystalline forms: α-crystal (pentahydrate, C_14_H_13_N_8_NaO_4_S_3_·5H_2_O), β-crystal (sesquihydrate, C_14_H_13_N_8_NaO_4_S_3_·1.5H_2_O), γ-crystal [C_14_H_13_N_8_NaO_4_S_3_·(HOCH_2_)_2_], and amorphous (Pikal and Delleman, 1989; Hu et al., 2002; Jacob et al., 2011). Interconversion occurs between these forms under certain conditions (Kamat et al., 1988; Osawa et al., 1988; Yang and Hu, 2005). The crystal structure of α-CEZ-Na was identified in 1983 (Stephenson and Diseroad, 2000) and manufactured by Fujisawa Co. Ltd., Japan; it was later transferred to Kyongbo Pharm. Co., Ltd., South Korea. Hu et al. identified another new form of α-CEZ-Na, which they obtained by using an isopropyl alcohol–water system (Hu et al., 2008); this was introduced to the Chinese market by Shenzhen Gosun Pharmaceutical Co., Ltd. These two α-CEZ-Na forms are similar: two symmetry-independent molecules are present in an asymmetric unit, where both molecules are well-ordered in the lattice, with large solvent tunnels. The thermogravimetric analysis (TGA) profiles are similar; however, their powder X-ray diffraction (PXRD) patterns (Figures S1, S2 in the electronic supplementary material) have certain differences. This indicates that the different manufacture processes affect the crystallization behavior of α-CEZ-Na, which results in different polymorphic forms.

The quality target product profile (QTPP) incorporates the intended quality attributes of the drug product to ensure efficacy and safety. The QTPP can be used as an effective tool to define the critical characteristics of the drug product and manufacturing process and thus ensure the intended performance of the product (Yu, 2008). Nuclear magnetic resonance (NMR) spectroscopy is a technique that exploits the magnetic attributes of nuclei and has been used to investigate the atomic environments of molecules. Solid-state NMR (SS-NMR) can provide detailed information on not just molecular structures but also intra- and intermolecular interactions, particularly molecular dynamics (Brown and Spiess, 2001). Thus, SS-NMR measurements are extremely useful for characterizing the crystal forms of pharmaceutical solids (Harris, 2007; Bai et al., 2010). It has been employed to characterize two amorphous forms and provide insight into the structures and molecular mobility of their amorphous forms (Frederick et al., 2013), detect interactions between the drug and substrate excipients (Remenar et al., 2004; Skotnicki et al., 2016), and gain information about the conformations of APIs and the interactions with water molecules (Chen et al., 2000). In this study, SS-NMR was combined with theoretical calculation to reveal the differences in the polymorphic structures of α-cefazolin sodium from different vendors.

## Experimental section

### Reagents and materials

CEZ-Na pentahydrate 1 (C_14_H_13_N_8_NaO_4_S_3_·5H_2_O, batch no: L100100, L100200, L100300) was obtained from Fujisawa Co., Ltd., Kirihara-cho, Fujisawa-shi, Kanagawa, Japan. CEZ-Na pentahydrate 2 (C_14_H_13_N_8_NaO_4_S_3_·5H_2_O, batch no: 1368, 1391, 1401) was obtained from Kyongbo Pharm. Co., Ltd., Silok-ro, Ahsan-si, Choongchungnam-do, South Korea. CEZ-Na pentahydrate 3 (C_14_H_13_N_8_NaO_4_S_3_·5H_2_O, batch no: 1203283, 1203423, 1203403) was obtained from Shenzhen Gosun Pharmaceutical Co., Ltd., Guangdong, China. CEZ-Na sesquihydrate was prepared by crystal transformation of pentahydrate as described previously (Yang and Hu, 2005). Amorphous cefazolin sodium was prepared from the powder for injection produced by the freeze-drying technique. The purity of all samples was >99.1%. All samples were verified by solution ^1^H and ^13^C NMR and used in all measurements without further purification.

### Powder X-ray diffraction

The PXRD patterns of the samples were recorded at room temperature (298 K) on a SmartLab diffractometer (Rigaku Corporation, Tokyo, Japan) with Cu Kα radiation (1.54 Å) at 45 kV and 200 mA. PXRD scans were recorded in continuous mode with a step size of 0.02° and scan rate of 1° per minute over a 2θ range of 2–60°.

### Thermogravimetric analysis

TGA measurements were performed on a TAQ 500 (TA Instruments, Inc., New Castle, DE, USA). The α-CEZ-Na samples (3.40 mg) was weighed and analyzed at a heating rate of 20°C/min from 20 to 160°C and purged with nitrogen gas (25 mL/min).

### Solid-state nuclear magnetic resonance spectroscopy

All SS-NMR spectra were obtained on a Bruker AVANCE II WB400 NMR spectrometer at 298 ± 2 K by using a 4 mm double-resonance (HX) probe at a ^13^C frequency of 100.61 MHz. The ^13^C chemical shifts were referenced externally to tetramethylsilane (TMS) of 0 ppm. The ^13^C CP/MAS experiments were carried out at a sample spinning rate of 10,000 ± 2 Hz with 4 mm BRUKER rotors and a contact time of 2 ms. The TPPM decoupling during data acquisition was provided by a proton decoupling field of 104.2 kHz. The recycle delays and number of scans of ^13^C CP/MAS experiments were 29 s and 320, respectively.

Two-dimensional ^1^H-^13^C heteronuclear correlation (HETCOR) experiments were performed with a Lee–Goldburg ^1^H-^1^H decoupling during the evolution period, which consisted of a 2.3 μs π/2 ^1^H pulse and four Lee–Goldburg cycles per evolution increment. Different contact times were optimized and used to assign all chemical shifts of the complex ^13^C spectrum. The short contact-time HETCOR experiment (i.e., 50 μs) provided correlations between directly bonded nuclei, and the long contact-time experiment (i.e., 300 μs) provided possible long-range correlations. Eighty evolution points were collected for the indirect proton dimension, each with 34 scans. The ^1^H dimension was referenced internally to the methyl group of CEZ-Na at 2.73 ppm. All reported proton shifts were scaled by 0.578, which is the expected scaling factor for Lee–Goldburg homonuclear decoupling. The data were processed by multiplication by a cosine-square function in the t_1_ and t_2_ dimensions prior to the Fourier transformation.

### Fourier transform infrared spectroscopy

Fourier transform infrared (FTIR) spectra were recorded on an EQUINOX 55 spectrophotometer (Bruker, Zurich, Switzerland). Spectra were recorded at 26°C from 4,000 to 400 cm^−1^ with a resolution of 1 cm^−1^. Measurement samples were prepared by grinding α-CEZ-Na powder with KBr and compressing the mixture into thin pellets. Temperature-programmed IR was conducted as follows. (a) Method 1: IR spectra were recorded at 26, 30, 35, 40, 45, 50, 55, 60, 65, 70, 75, 80, 85, 90, 95, 100, and 105°C at a heating rate of 5°C/min from 26 to 105°C. (b) Method 2: IR spectra were recorded at 50, 55, 56, 58, 60, 62, 64, 65, 66, 68, 70, 71, 72, 73, 74, 75, 76, 77, and 80°C at a heating rate of 0.5°C/min from 26 to 80°C.

### Molecular conformational analysis

Conformational analysis was carried out by using the CONFORMER module of BIOVIA Material Studio with the COMPASS II force field. A torsion energy contour map was built up for the selected compounds, where the selected torsion angles were varied from 0° to 360° with a step size of 60° and constrained at each grid point whilst all other degrees of freedom were optimized for the molecule.

The cell structures were optimized by using the Forcite module of BIOVIA Materials Studio with the COMPASS II force field.

## Results and discussion

### NMR data for cefazolin

**Figure 2:**
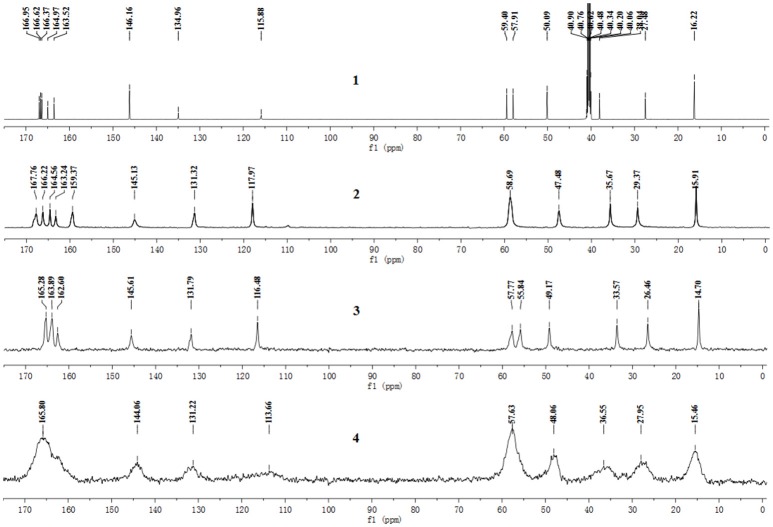
^13^C NMR spectra of CEZ-Na: (**1**) ^13^C NMR data in DMSO-*d*_6_, (**2**) ^13^C SS NMR spectra of α-CEZ-Na **1**, (**3**) ^13^C SSNMR spectra of β-CEZ-Na, and (**4**) ^13^C SSNMR spectra of amorphous CEZ-Na.

The pentahydrate, sesquihydrate, and amorphous forms of CEZ-Na were distinctly distinguished by ^13^C SS-NMR spectral patterns with better resolved signals than those reported previously (Byrn et al., 1985). The carbon nuclei of the pentahydrate and sesquihydrate exhibited single peaks with no splitting (Figure 2) as compared with that of the amorphous form, which has broad resonances due to the distribution of conformers. Furthermore, comparison of the NMR data of pentahydrate and sesquihydrate showed that the chemical shifts and number of resonances for β-lactam (55–58 ppm) and carbonyl group (158–170 ppm) of the pentahydrate were distinctly different from those of the sesquihydrate.

The ^13^C NMR data obtained from CEZ-Na pentahydrate in inert DMSO-*d*_6_ was typically similar to the data obtained from SSNMR. Hence, the data assigned from ^13^C NMR served as guidance for the assignment of the ^13^C SS-NMR data. Distortionless enhancement of polarization transfer (DEPT), ^1^H–^1^H homonuclear correlation spectroscopy (COSY), heteronuclear single quantum coherence (HSQC), and heteronuclear multiple bond correlation (HMBC) were explored for accurate assignment of all the signals, especially the ambiguous signals at 163.5, 165.0, 166.4, 166.6, and 167.0 ppm. In particular, by the correlations between H6 and C8; H7 and C8, and C19; H10 and C12; H_3_17 and C14; H20 and C19 and C25. Thus, all protons and carbons were assigned. The assignments of C12, 14, and C19 were corrected (Tori et al., 1981; Figures S3-1–S3-4 and Table 1).

**Table 1:** NMR Data (δ) for cefazolin sodium pentahydrate.

**Peak No**.	**^13^C NMR in DMSO-*d*_6_**	**SS-NMR**
2	135.0	131.3
3	115.9	118.0
4	27.5	29.4
6	57.9	58.7
7	59.4	58.7
8	163.5	158.4
9	165.0	163.2
10	38.0	35.7
12	167.0	164.6
14	166.4	166.2
17	16.2	15.9
19	166.6	167.8
20	50.1	47.5
25	146.2	145.1

**Figure 3:**
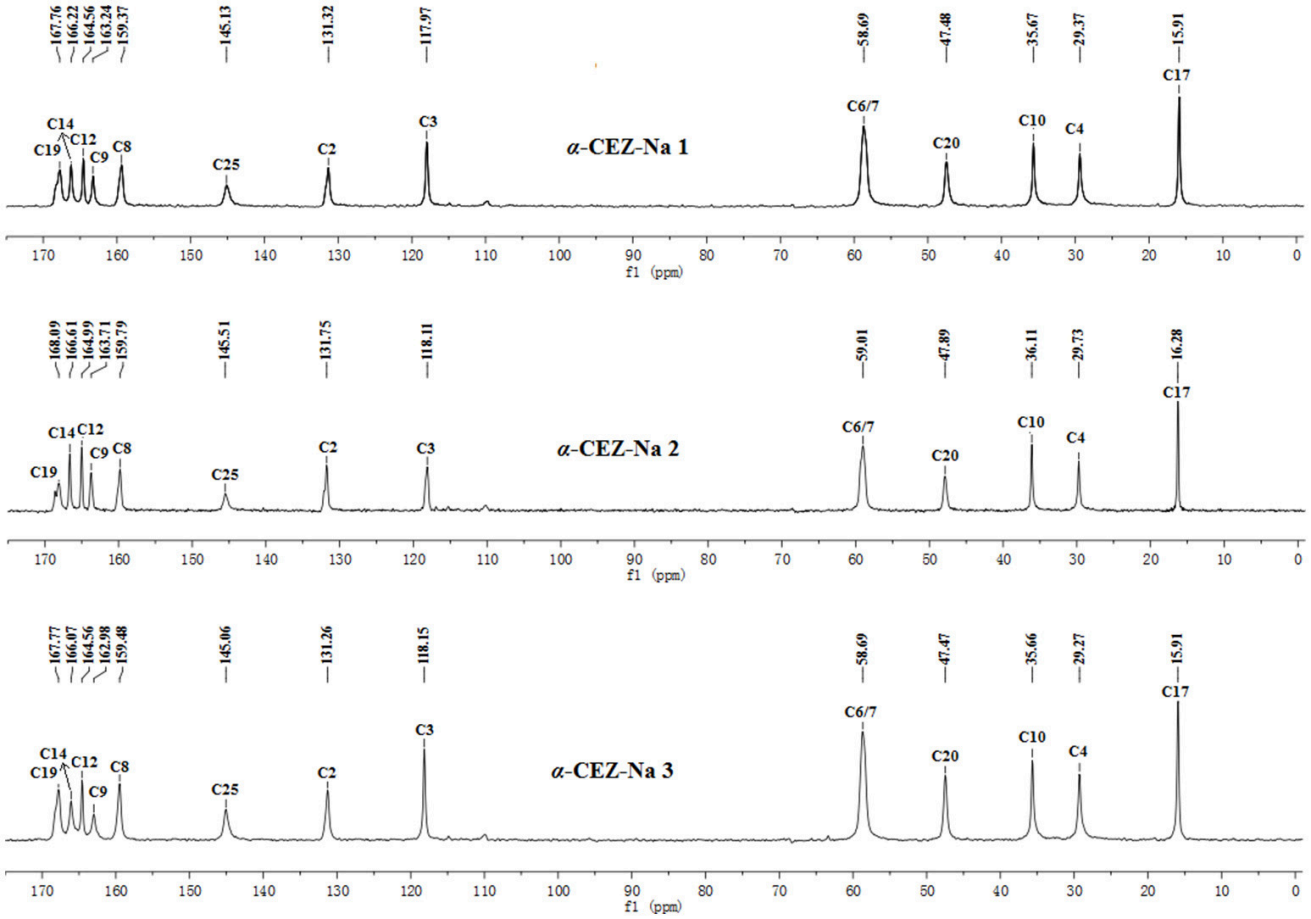
^13^C SSNMR spectra of α-CEZ-Na produced by different pharmaceutical companies.

**Figure 4:**
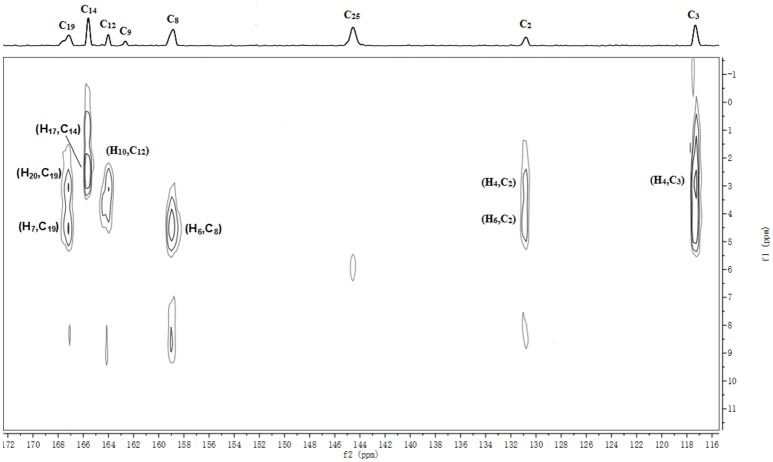
Section of the ^1^H-^13^C HETCOR spectra of α-CEZ-Na taken with a contact time of 300 μs.

The batch-to-batch consistency of the ^13^C SS-NMR data for the three forms of CEZ-Na pentahydrate 1, 2, and 3, respectively, indicated the stability of the manufacturing process and the reproducibility of the method (Figures S4–S6 in the electronic Supplementary Material). Distinct differences among CEZ-Na pentahydrate 1, 2, and 3 were observed with respect to the shape of peak C19 (i.e., intensity of the shoulder peak of C19) and the relative intensity of peak C14 (Figure 3). The ^1^H–^13^C HETCOR spectra were recorded at two contact times (50 and 300 μs) so that all chemical shifts observed in the complex ^13^C NMR spectrum of CEZ-Na could be assigned. The short contact-time sequence provided correlations between directly bonded nuclei, while the long-contact-time sequence provided possible long-range correlations, such as the dipolar coupling of the hydroxyl proton to the carbon to which the hydroxyl group is bonded (Figures S7–S8 and Figure 4). All carbon chemical shifts of CEZ-Na were assigned based on the significant correlations observed in the ^1^H–^13^C HETCOR spectra (300 μs) between H-4/C-6 and C-10; H-7/C-19; H-10/C-12; H_3_-17/C-14; H-20/C-19, and H-25/C-20 as well as the data obtained from solution NMR. Thus, all carbon chemical shifts of CEZ-Na were assigned (Table 2). Differences in signals among CEZ-Na pentahydrate 1, 2, and 3 were further ascertained at C14 and C19; this may be attributed to the minor configurational differences in the packing of molecules in a unit cell (Bai et al., 2010; Wang et al., 2012).

**Table 2:** The assignment of IR data.

**Wavenumbers(cm^−1^)**	**Band assignment**	**Wavenumbers(cm^−1^)**	**Band assignment**
3,431	ν_OH_(_crystalwater_)	1,601	ν_as_(COONa)
3,291	ν_NH_(CONH)	1,544	ν_NH_(CONH Iband)
1,761	ν_CO_(β-lactamC = O)	1,385	δ_CH_(CH_3_)
1668	ν_NH_(CONH I band)	1,360	ν_CO_(COONa)
		1,285–1,003	ν_CN_(CONH, C-N)

### FTIR data for cefazolin

Different solid-state packing interactions provide small wavenumber shifts, which can be assessed if high-resolution vibrational measurements are applied (Arrais et al., 2003; Arrais and Savarino, 2009). The IR spectra of CEZ-Na pentahydrate 1, 2, and 3 were examined at 26°C with a high-resolution of 1 cm^−1^, and the results were undistinguished. Compared to the spectra reported in the literature (Pedroso and Salgado, 2013), no significant differences were found (Table 2 and Figure S9 in the electronic Supplementary Material). The IR spectral trends of pentahydrate 1, 2, and 3 were examined and analyzed in the range of 26–105°C (method 1). The general trends of the IR spectrum significantly changed at 75°C: [ν_OH(crystal water)_ and ν_NH(CONH)_] were shifted by Δν of +17 and +11 cm^−1^, respectively, whereas ν_CO(β−*lactam C* = *O*)_ was shifted by Δν of −9 cm^−1^. In addition, the decreasing intensity of the ν_NH(CONH I band)_ was replaced by the increasing intensity of the absorption at 1,689 cm^−1^. This indicated changes in the intermolecular interactions of water molecules at approximately 75°C (Figure S10 in the electronic Supplementary Material). For further investigation of the differences between pentahydrate 1, 2, and 3 and the effect of temperature on the crystal form of CEZ-Na, a more detailed comparison was made (method 2). During the characteristic stage of the decreasing intensity of the ν_NH(CONHI band)_, pentahydrate 1 clearly changed at 76°C, and pentahydrate 2 and 3 accordingly changed at 71°C.

## Discussion

The different physical and chemical properties of a drug substance with polymorphic forms result in different values for the chemical reactivity, dissolution rate, melting point apparent solubility, vapor pressure, optical and mechanical properties, and density. These characteristics not only decide how or if a drug substance can be manufactured, but also affect the bioavailability, stability and dissolution of drug products. Therefore, the safety, efficacy, and quality of a drug product are largely depend on the polymorphism of its drug substance.

In conformational polymorphism, a molecule in an asymmetric unit can adopt different conformations through a controlled crystallization process. This is a critical part of polymorphism. Because SS-NMR is sensitive to variations in internuclear distances and local electronic structures resulting from various conformations, it is a powerful probe for characterization. In this study, the similarity of the TGA, FTIR, and SS ^13^C NMR spectra indicated a similar water content and crystal structure for CEZ-Na pentahydrate 1, 2, and 3. Two symmetry-independent molecules are present in an asymmetric unit, and both molecules are well-ordered in the lattice with large solvent tunnels that were identified by single-crystal XRD. XRD is a common experimental method for obtaining a detailed crystal structure in the ideal state. Thus, it failed to provide the integral solid state of the API and falsely indicated the presence of only a single structure for the polymorph. Although two independent molecules are present in an asymmetric unit of CEZ-Na pentahydrate, the symmetry of the two molecules should lead to single peaks without splitting in the ^13^C SS NMR spectra. All signals were displayed as single peaks, except the C-19 resonances; this indicates that molecules present in an asymmetric unit may adopt different conformations (Mimura et al., 2002; Liu et al., 2005).

**Figure 5:**
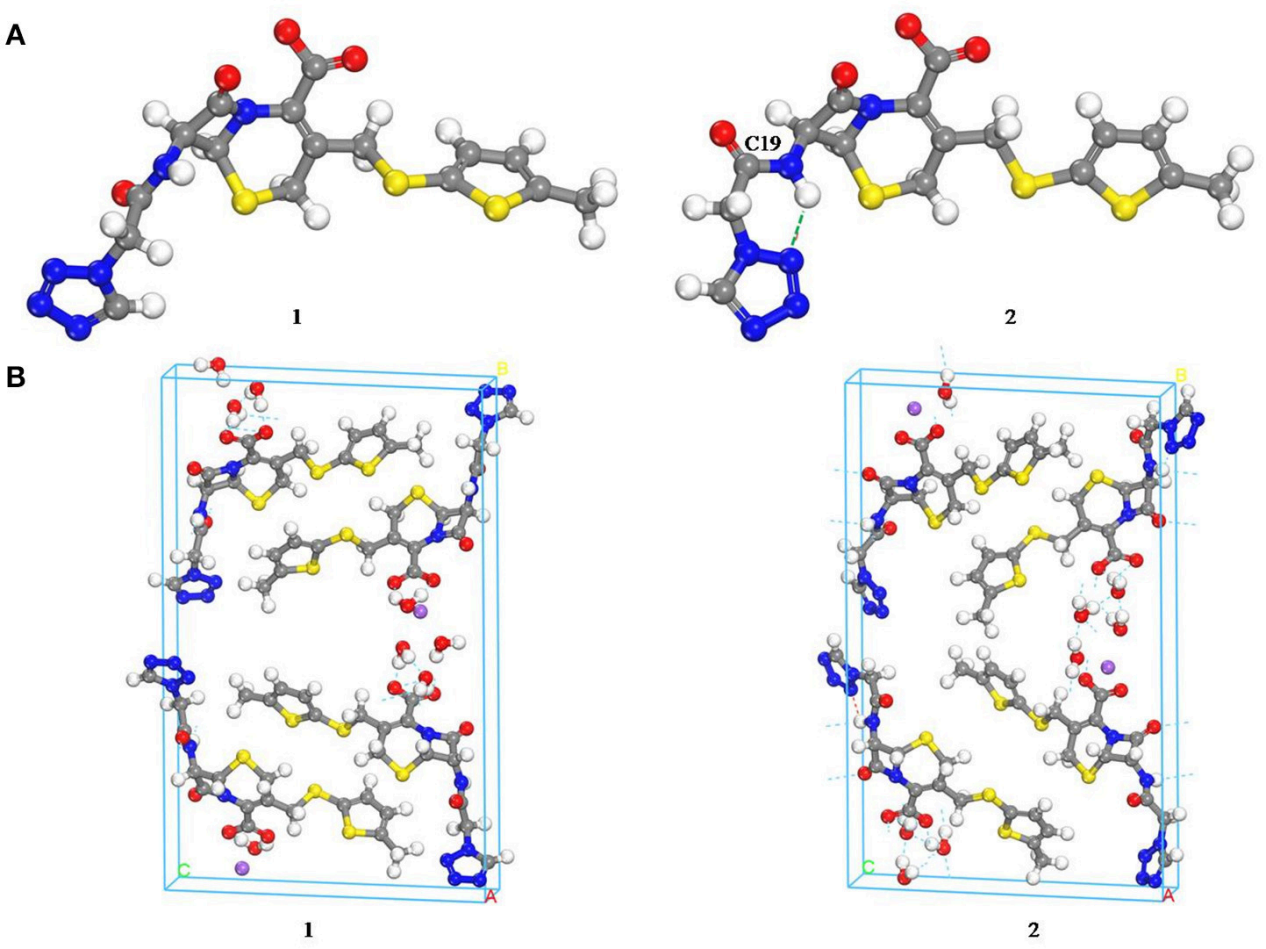
Comparison of the molecular conformations of CEZ-Na **(A)** the conformations of α-CEZ-Na: 1. from crystal structure of α-CEZ-Na; 2. from calculation of molecular conformational analysis; **(B)** Global molecular arrangements: 1. from crystal structure of α-CEZ-Na; 2. from calculation of molecular conformational analysis).

Computational chemistry methods were utilized to study the conformation of CEZ-Na for exploring the differences with conformational polymorphism (Figure 5). The initial conformation **1** was reported by us earlier (Hu et al., 2008). The geometry of the conformation was energy-minimized by the CONFORMER module of the BIOVIA Material Studio with the COMPASS II force field to obtain conformation **2**. The cell structures were optimized by using the Forcite module of BIOVIA Materials Studio with the COMPASS II force field. Similar crystal-lattice energies were generated (**1**: −817.8 kcal/mol; **2**: −830.1 kcal/mol), which indicates that conformational polymorphism exists in CEZ-Na. The shoulder peak of C19 seen in SS-NMR data may be related to intramolecular hydrogen bond formation as seen in conformation 2. Although the intermolecular arrangements are quite different and should also affect C20 and C25, no significant change was found for the resolution of SSNMR.

Marginal differences were observed among CEZ-Na pentahydrate 1, 2, and 3 produced by different pharmaceutical companies, with PXRD, ^13^C SS-NMR spectra, temperature-programmed IR, and high-resolution TGA observations (Figure S11 in the electronic Supplementary Material) displaying subtle differences in the crystal structure of the pentahydrates. In particular, it was speculated that the shape of peak C19 (intensity of shoulder peak of C19) and the relative intensity of peak C14 were influenced by the proportion of conformation **2** differing among pharmaceutical companies (the proportion was greatest for CEZ-Na 2).

Understanding the solid-state structure of API at the local and long-range levels is crucial to developing strategies for the proper administration of these drugs. SS-NMR spectra and X-ray crystallography are the two techniques of choice to investigate the solid-state structure of synthetic pharmaceuticals and natural products. SS-NMR supplies structural information on powder amorphous solids for which single-crystal diffraction structures cannot be obtained. And SSNMR is uniquely sensitive to the local structure in polymorphic systems. Integrating all of these methods, such as TG, XRPD, and IR can provide insight into the local and long-range structure of polymorphic materials.

## Conclusions

By comparing and analyzing of the SS-NMR data, it was speculated that conformation **2** was exhibited in three forms of CEZ-Na pentahydrate, and CEZ-Na pentahydrate **2** exhibited the highest percentage of conformation **2**. These differences with respect to the percentage of conformation **2** in the molecular packing in the unit cell were attributed to a range of manufacturing processes, such as drying, wet granulation, spray-drying, and compaction. Future study will involve investigating the consistency in quality and the relationship between minor differences of polymorphs and the stability and efficacy.

The Food and Drug Agency (FDA) defines QTPP as the quality attributes related to the safety and efficacy of a product. After QTPP has been ascertained, the identification of the relevant critical quality attributes (CQAs) to ensure the intented product quality is the next important work. Understanding the solid form of an active pharmaceutical ingredient (API) is a key part of its development. So understanding the differences in the polymorphic structure of α-cefazolin sodium may help with making modifications to incorporate new knowledge with the product's development.

## Author contributions

YT: design of experiments, analysis of experimental data; WW: SS-NMR data acquisition; W-BZ: PXRD and TGA data acquisition; J-QQ: theoretical study on molecular conformational analysis; C-QH: design of experiments, analysis of experimental data.

### Conflict of interest statement

The authors declare that the research was conducted in the absence of any commercial or financial relationships that could be construed as a potential conflict of interest.
