# Supplementary material for: Application of Solid-State NMR to Reveal Structural Differences in Cefazolin Sodium Pentahydrate From Different Manufacturing Processes
Source: Front Chem. 2018 Apr 10;6:113. doi: 10.3389/fchem.2018.00113 (PMC5902681; doi:10.3389/fchem.2018.00113)
Supplement: Supplementary file 1 [file Presentation1.pdf]

Application of Solid-State NMR to Reveal Structural Differences in Cefazolin Sodium Pentahydrate from Different Manufacturing Processes

Ye Tian[1] • Wei D Wang [2] • Wen-Bo Zou[1] • Jian-Qin Qian[3] • Chang-Qin Hu[1*]

1. *National Institute for Food and Drug Control, Chongwen District, Beijing 100050, Republic of China*

2. *State Key Laboratory of Applied Organic Chemistry, College of Chemistry and Chemical Engineering, Lanzhou University, Lanzhou,Gansu, 730000, China*

3. *Zhejiang Institute for Food and Drug Control, Binjiang District, Hangzhou, Zhejiang, 310052, Republic of China*

Supplementary Content

* Corresponding author. Tel.: +86 10 67095308; fax: +86 10 65115148.

E-mail address: hucq@nicpbp.org.cn (C.-Q. Hu)

List of Contents

No.	Content	Page
1	**Figure S1.** X-Ray powder diffraction spectra of *α*-CEZ-Na **1-3**	S3
2	**Figure S2.** TGA profiles of *α*-CEZ **1-3**	S3
3	**Figure S3-1.** ^{1}H NMR spectra of *α*-CEZ-Na **in** DMSO-d_6	S4
4	**Figure S3-2.** ^{13}C NMR spectra of *α*-CEZ-Na **in** DMSO-d_6	S5
5	**Figure S3-3.** HSQC spectra of *α*-CEZ-Na **in** DMSO-d_6	S6
6	**Figure S3-4.** HMBC spectra of *α*-CEZ-Na **in** DMSO-d_6	S7
7	**Figure S4.** Batch-to-batch consistency of ^{13}C SSNMR data for *α*-CEZ-Na **1**	S8
8	**Figure S5.** Batch-to-batch consistency of ^{13}C SSNMR data for *α*-CEZ-Na **2**	S9
9	**Figure S6.** Batch-to-batch consistency of ^{13}C SSNMR data for *α*-CEZ-Na **3**	S10
10	**Figure S7.** ^{1}H-^{13}CHETCOR spectrum for *α*-CEZ-Na taken (50 μs)	S11
11	**Figure S8.** Section of the ^{1}H-^{13}C HETCOR spectrum for *α*-CEZ-Na (300μs)	S12
12	**Fig. S9** IR spectra of *α*-CEZ-Na **1- 3**	S13
13	**Fig.S10** IR spectra of *α*-CEZ-Na **3** at 75℃	S13
14	**Fig. S11** The high resolution TGA profiles of *α*-CEZ-Na **1, 2** and **3**	S14

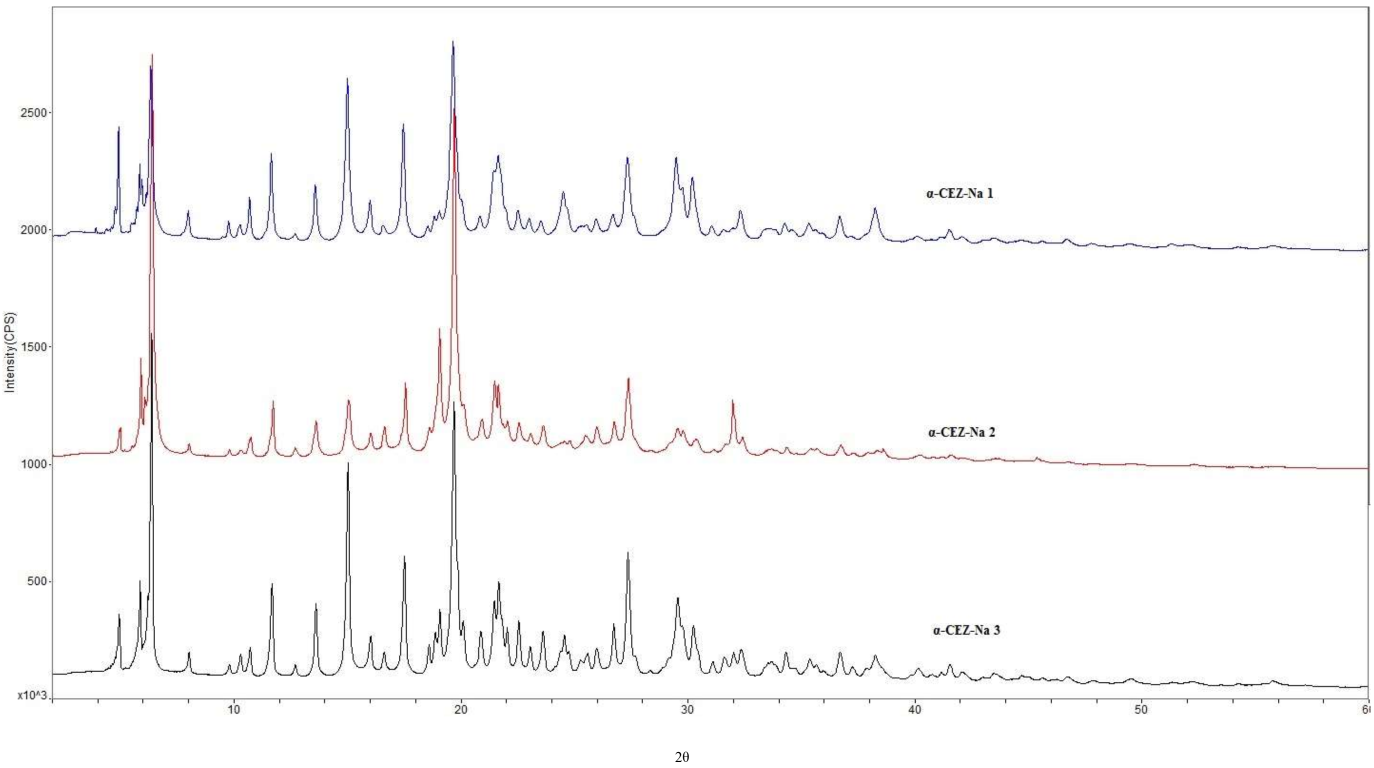

Fig. S1 X-Ray powder diffraction spectra of *α*-CEZ-Na **1-3**, showing the difference among *α*-CEZ-Na **1-3.**

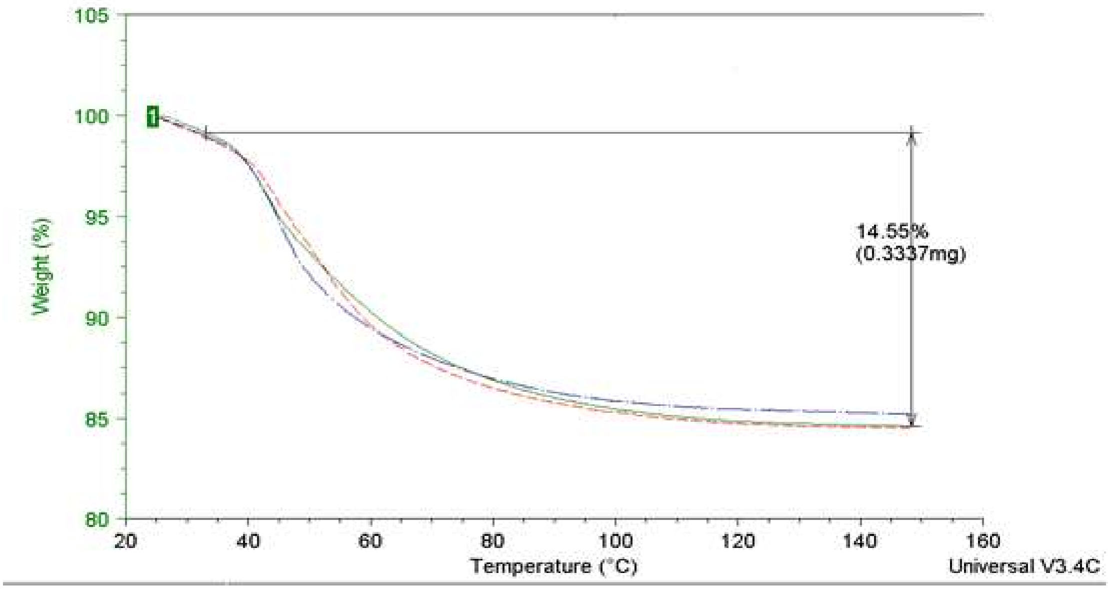

Fig. S2 TGA spectra of *α*-CEZ 1-3 (—*α*-CEZ 1; ---- *α*-CEZ 2; — *α*-CEZ 3) (water content:14.6%)

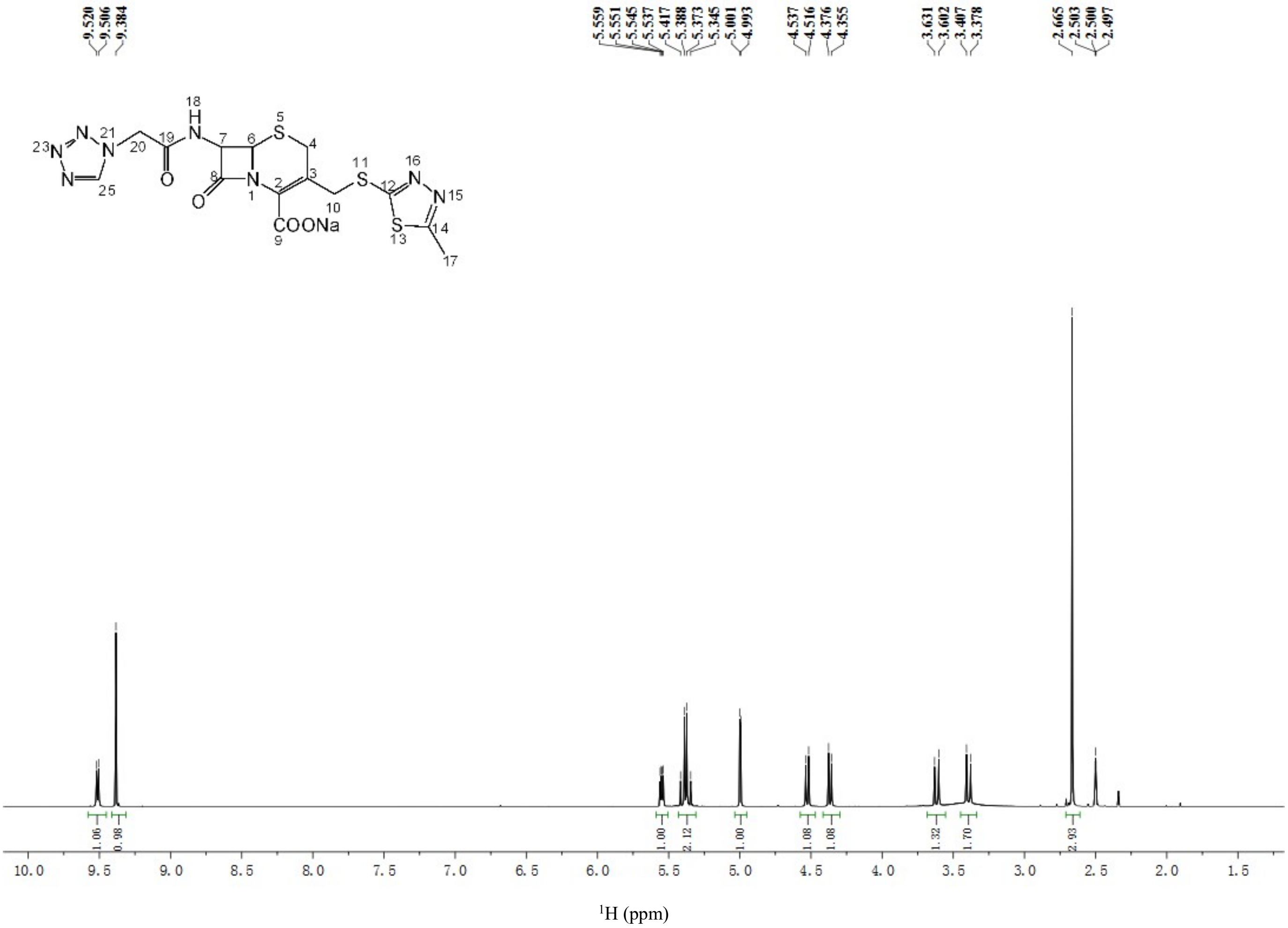

Fig. S3-1 ^{1}H NMR spectra of *α*-CEZ-Na **in** DMSO-d_6

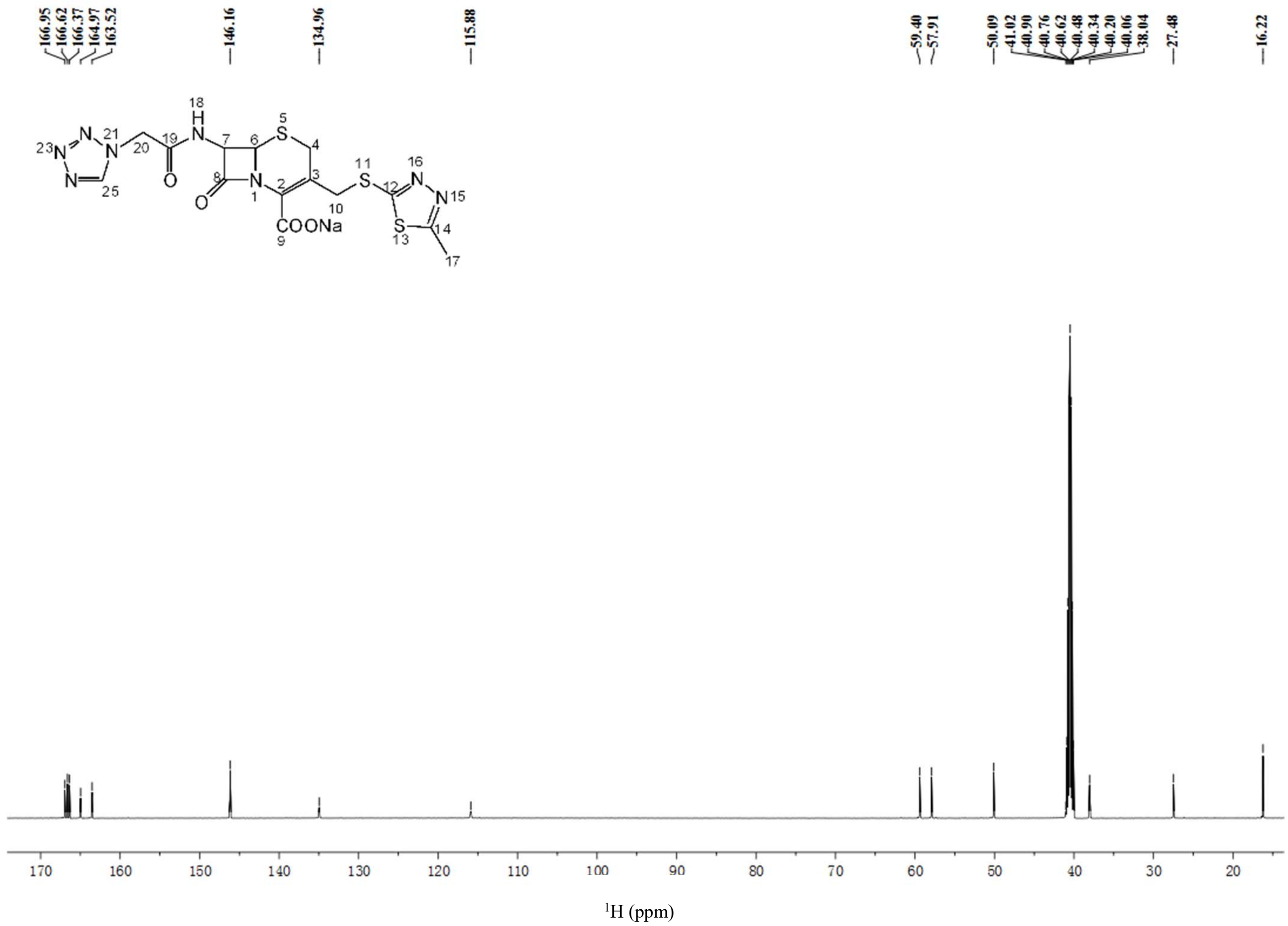

Fig. S3-2 ^{13}C NMR spectra of α-CEZ-Na **in** DMSO-d_6

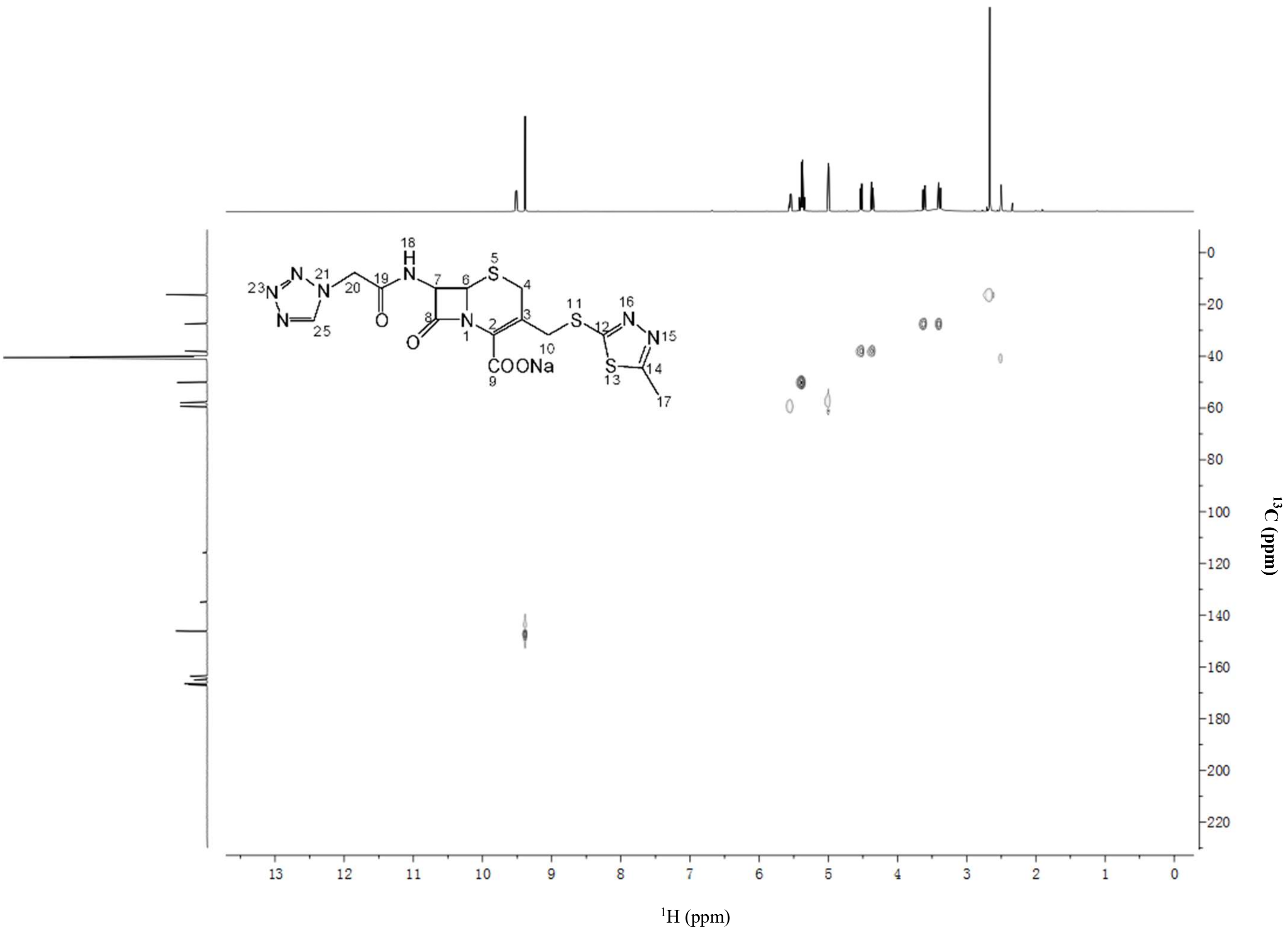

Fig. S3-3 HSQC spectra of α-CEZ-Na **in** DMSO-d_6

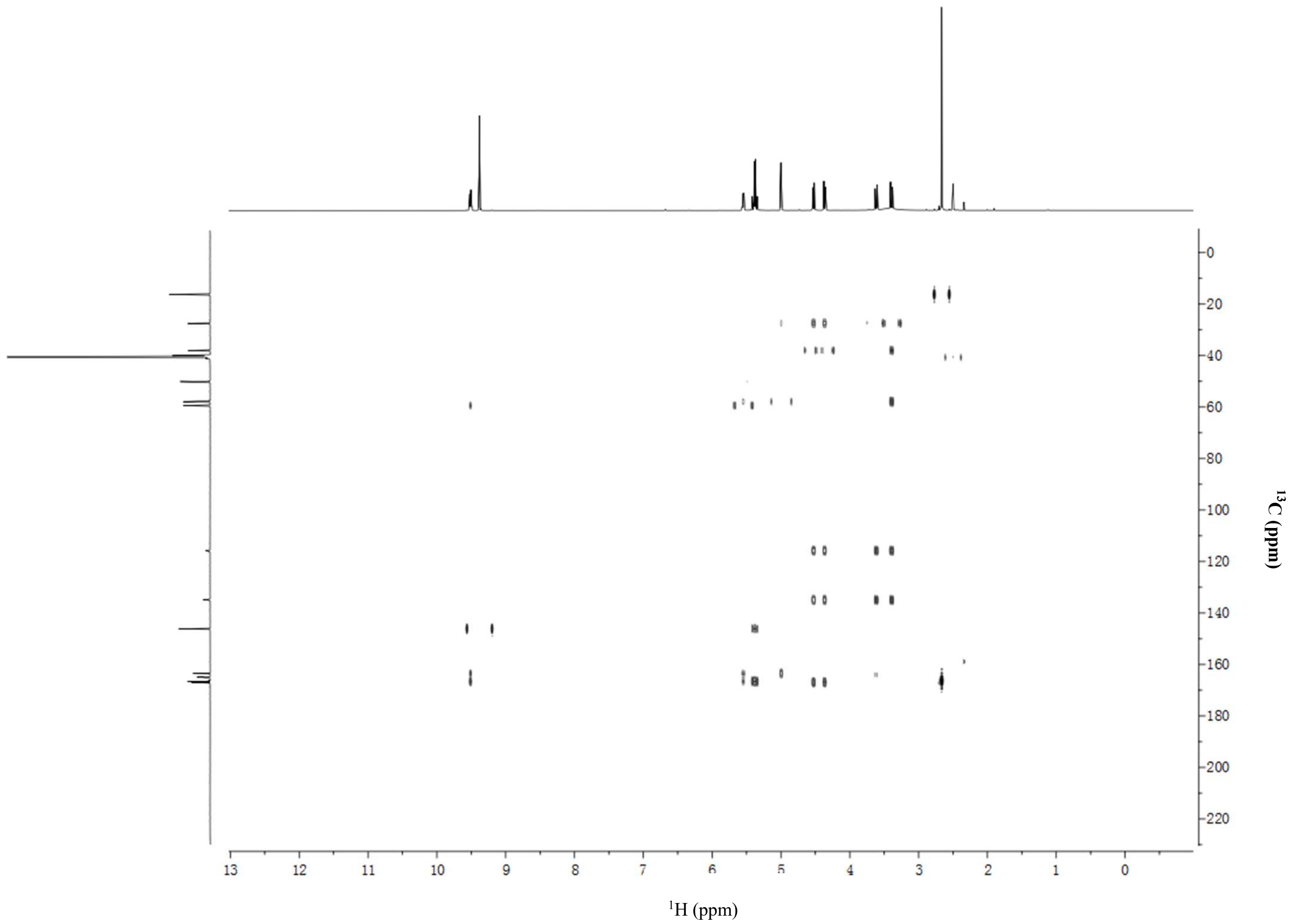

Fig. S3-4 HMBC spectra of *α*-CEZ-Na **in** DMSO-d_6

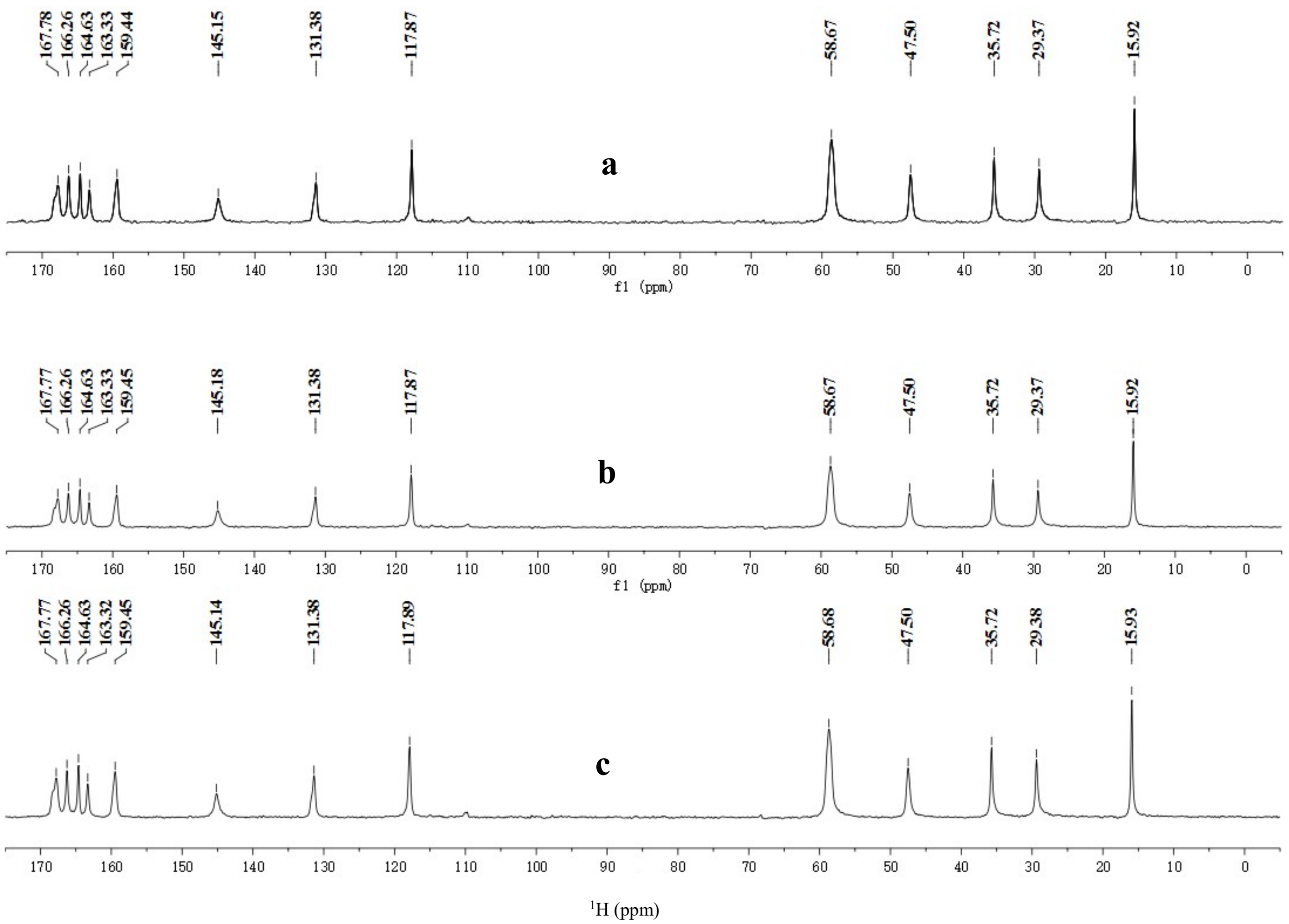

Fig. S4 Batch-to-batch consistency of ^{13}C SSNMR data for α-CEZ **1**(Batch a: L100100; Batch b: L100200; Batch c: L100300)

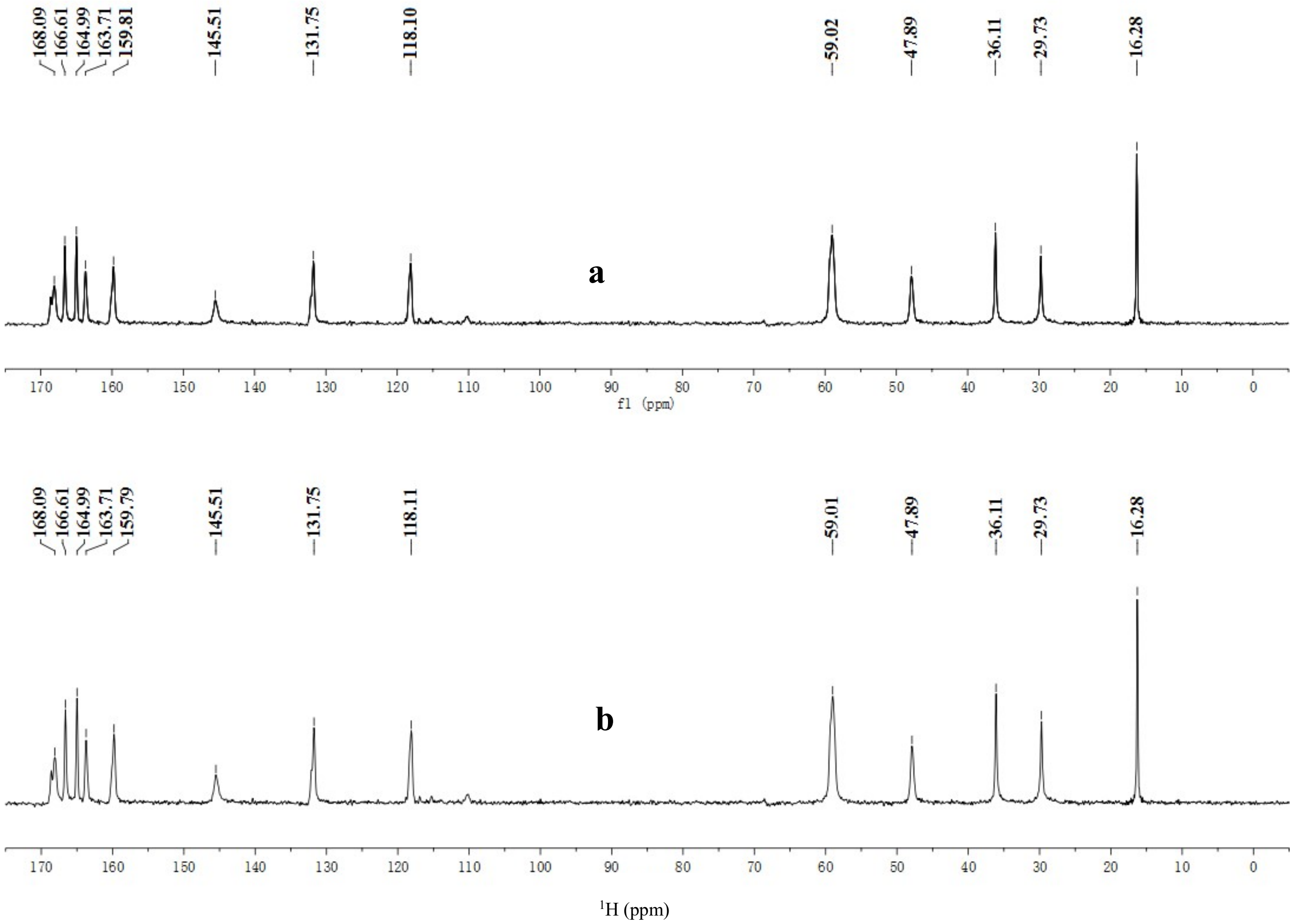

Fig. S5 Batch-to-batch consistency of ^{13}C SSNMR data for α-CEZ 2(Batch a: 1368; Batch b: 1401)

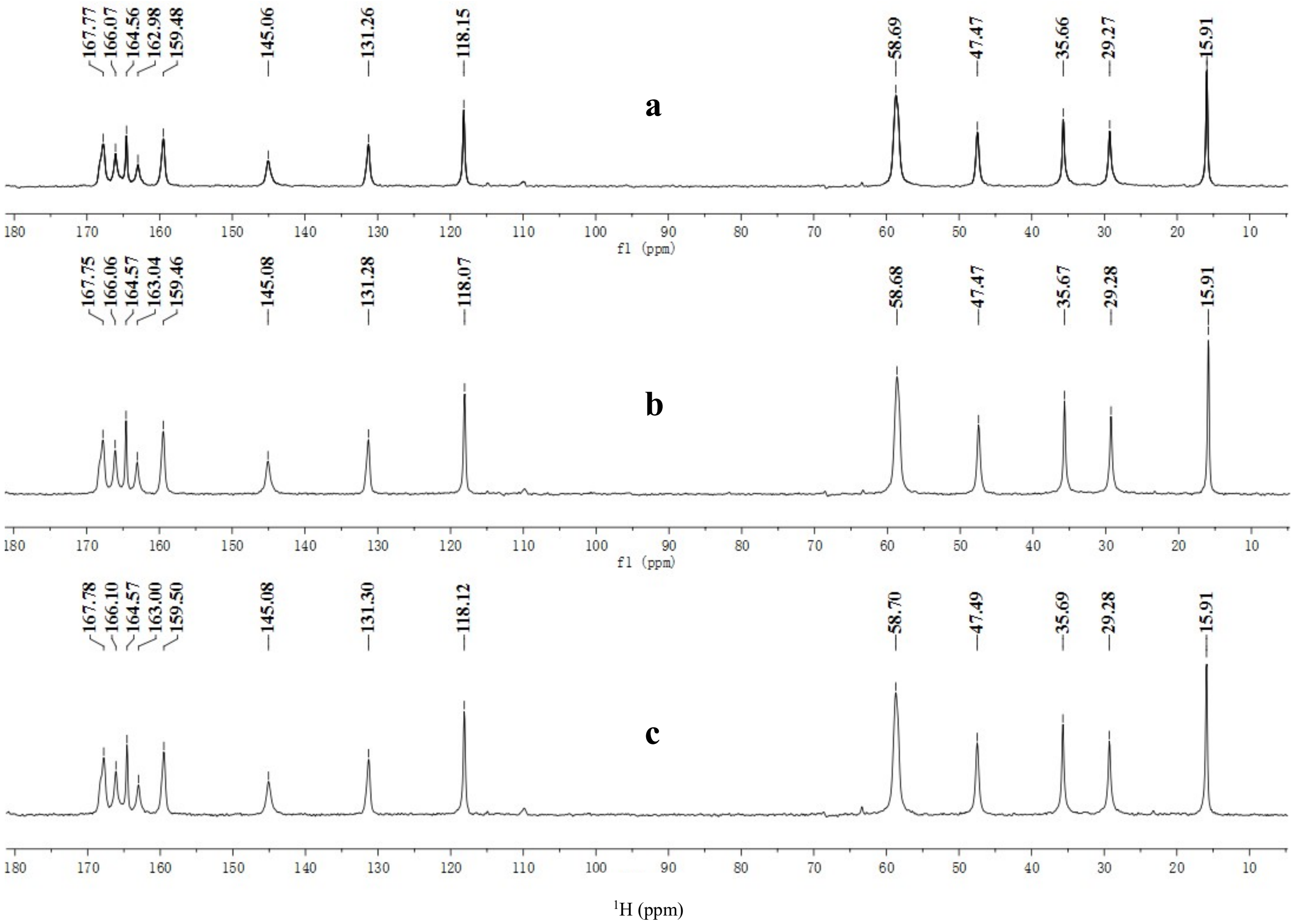

Fig. S6 Batch-to-batch consistency of ^{13}C SSNMR data for *α*-CEZ 3(Batch a: 1203283; Batch b: 1203423; Batch c: 1203403)

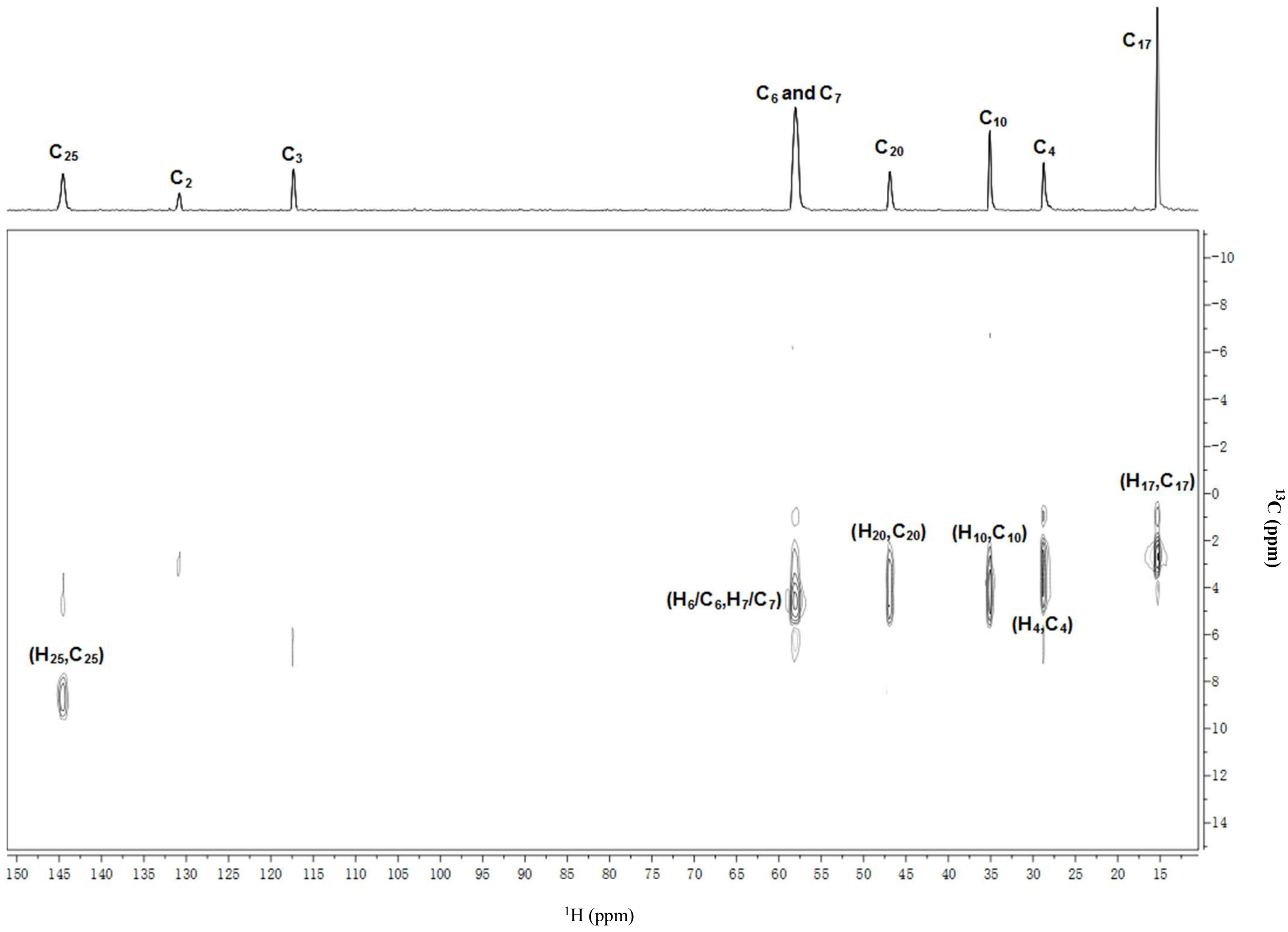

Fig. S7 ^{1}H-^{13}CHETCOR spectrum for CEZ-Na pentahydrate taken with a contact time of 50 μs

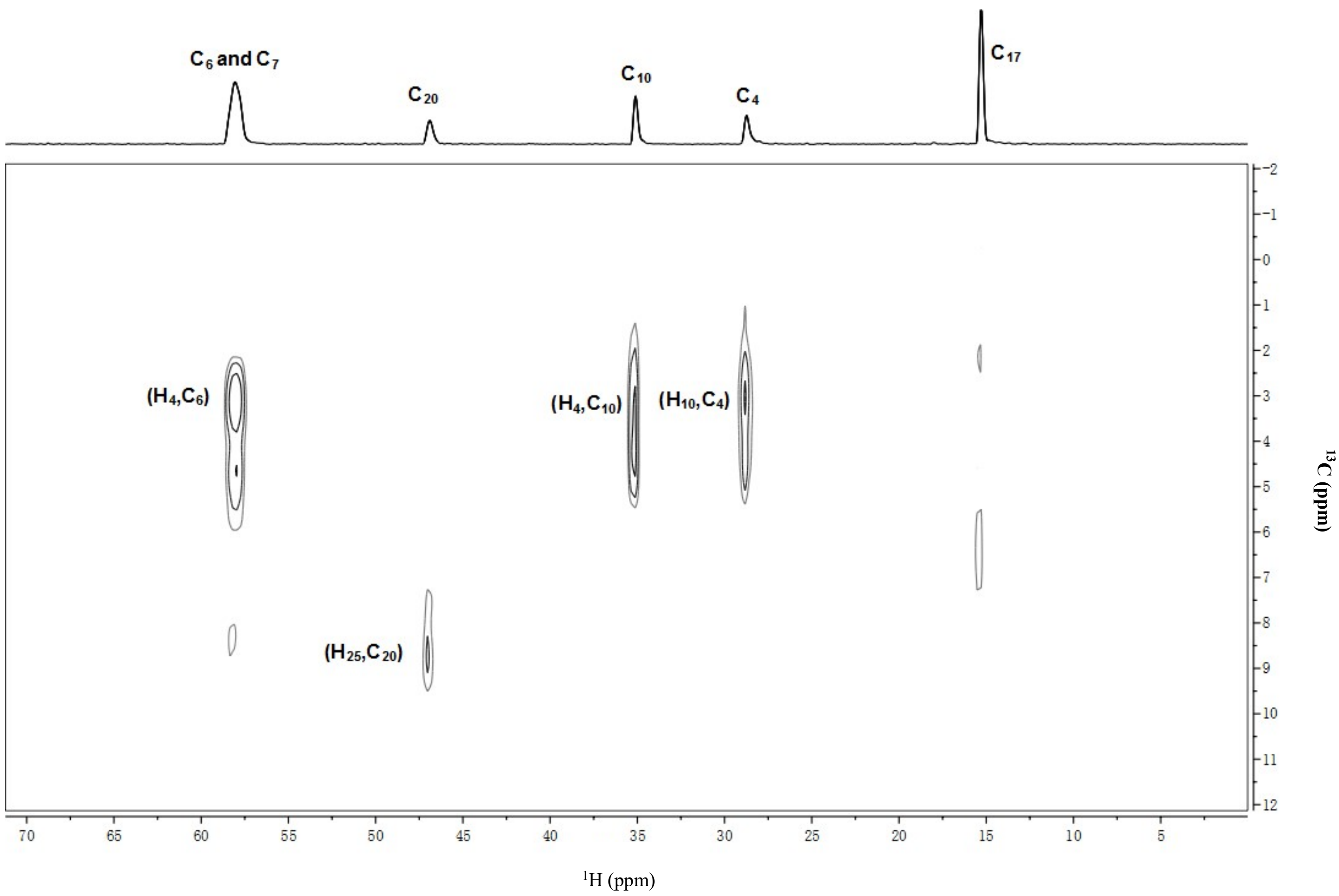

Fig. S8 Section of the ^{1}H-^{13}CHETCOR spectrum for CEZ-Na pentahydrate taken with a contact time of 300μs

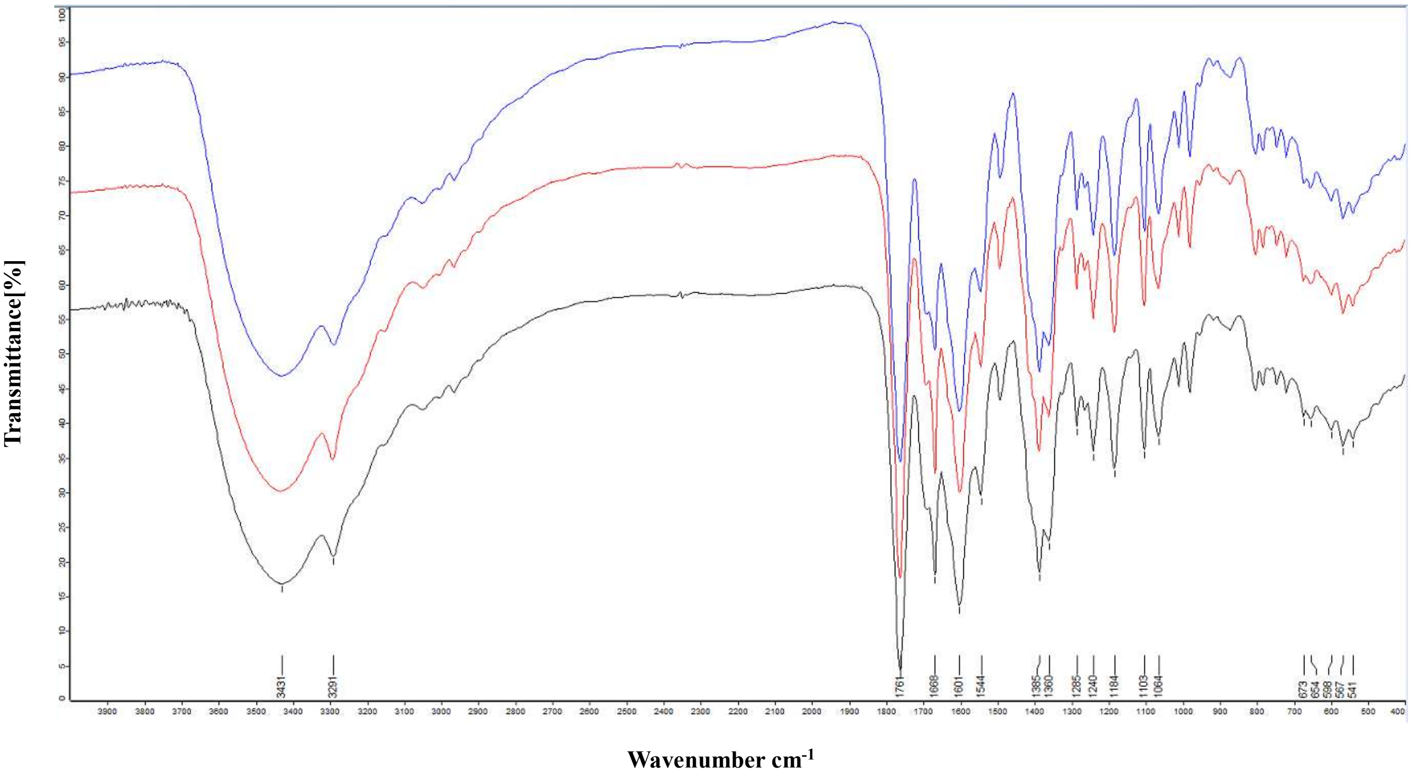

Fig. S9 IR spectra of α-CEZ-Na 1- 3(— α-CEZ 1; —α-CEZ 2; — α-CEZ 3)

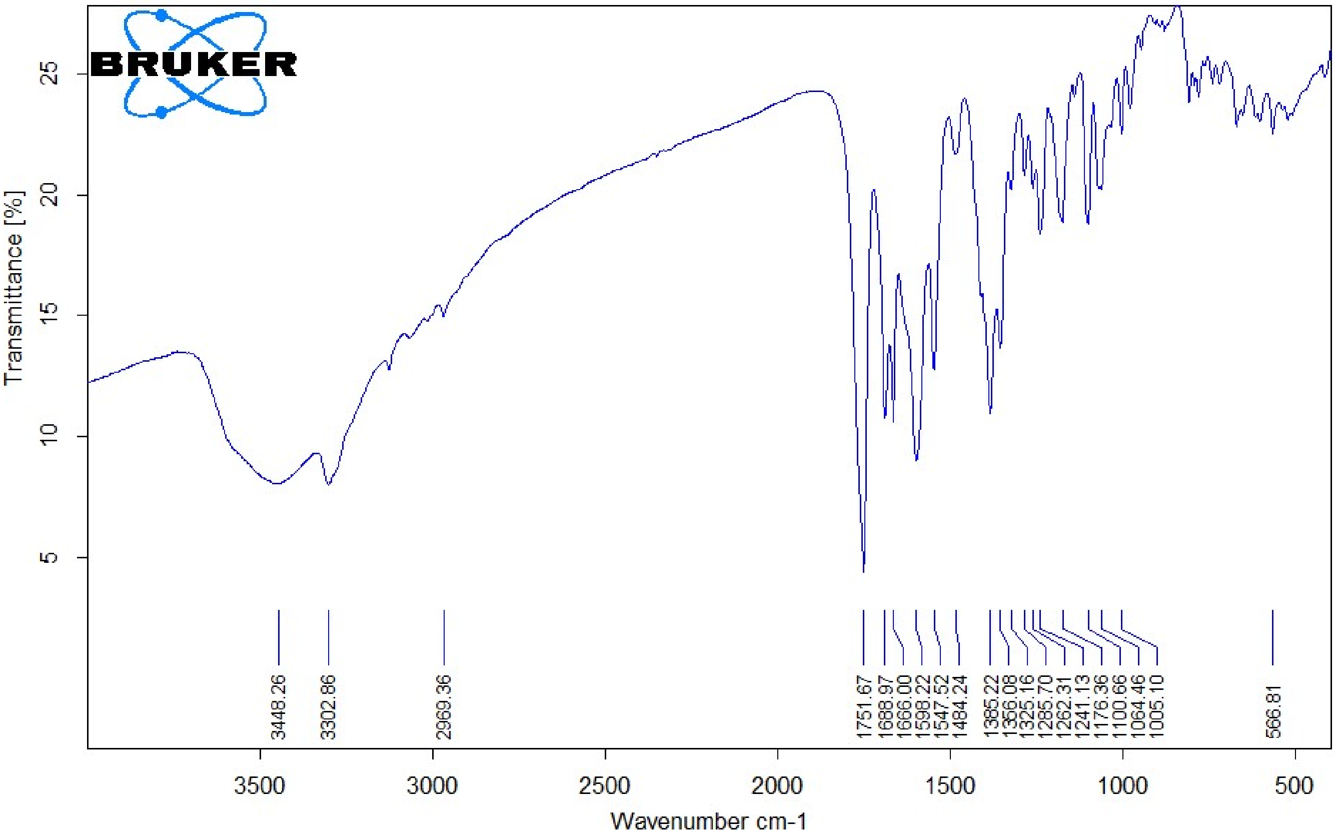

Fig. S10 IR spectra of α-CEZ-Na 3 at 75℃

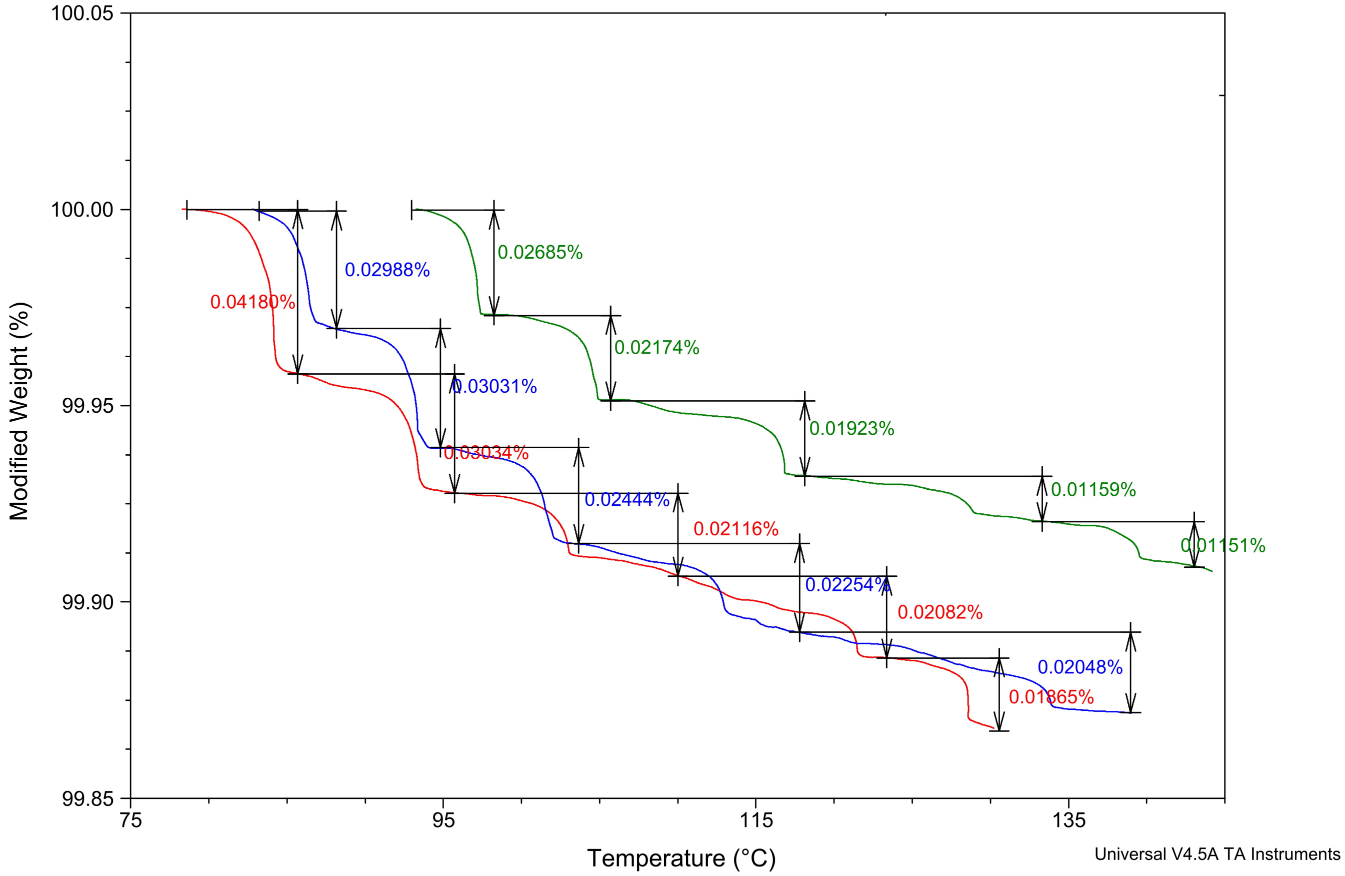

Fig. S11 The high resolution TGA spectra of *α*-CEZ-Na 1, 2 and 3 (━ *α*-CEZ 1;━ *α*-CEZ 2;━ *α*-CEZ 3)
